# Evidence Suggesting That *Francisella tularensis* O-Antigen Capsule Contains a Lipid A-Like Molecule That Is Structurally Distinct from the More Abundant Free Lipid A

**DOI:** 10.1371/journal.pone.0157842

**Published:** 2016-06-21

**Authors:** Jason H. Barker, Justin W. Kaufman, Michael A. Apicella, Jerrold P. Weiss

**Affiliations:** 1 Inflammation Program and Department of Internal Medicine, University of Iowa, Iowa City, IA, United States of America, and Veterans Affairs Medical Center, Iowa City, IA, United States of America; 2 Inflammation Program and Department of Microbiology, University of Iowa, Iowa City, IA, United States of America, and Veterans Affairs Medical Center, Iowa City, IA, United States of America; University of Louisville, UNITED STATES

## Abstract

*Francisella tularensis*, the Gram-negative bacterium that causes tularemia, produces a high molecular weight capsule that is immunologically distinct from *Francisella* lipopolysaccharide but contains the same O-antigen tetrasaccharide. To pursue the possibility that the capsule of *Francisella* live vaccine strain (LVS) has a structurally unique lipid anchor, we have metabolically labeled *Francisella* with [^14^C]acetate to facilitate highly sensitive compositional analysis of capsule-associated lipids. Capsule was purified by two independent methods and yielded similar results. Autoradiographic and immunologic analysis confirmed that this purified material was largely devoid of low molecular weight LPS and of the copious amounts of free lipid A that the Francisellae accumulate. Chemical hydrolysis yielded [^14^C]-labeled free fatty acids characteristic of *Francisella* lipid A but with a different molar ratio of 3-OH C18:0 to 3-OH C16:0 and different composition of non-hydroxylated fatty acids (mainly C14:0 rather than C16:0) than that of free *Francisella* lipid A. Mild acid hydrolysis to induce selective cleavage of KDO-lipid A linkage yielded a [^14^C]-labeled product that partitioned during Bligh/Dyer extraction and migrated during thin-layer chromatography like lipid A. These findings suggest that the O-antigen capsule of *Francisella* contains a covalently linked and structurally distinct lipid A species. The presence of a discrete lipid A-like molecule associated with capsule raises the possibility that *Francisella* selectively exploits lipid A structural heterogeneity to regulate synthesis, transport, and stable bacterial surface association of the O-antigen capsular layer.

## Introduction

*Francisella tularensis* is a Gram-negative bacterium capable of causing a life-threatening disseminated infection after exposure to only a few bacteria [1]. The initial infection induces only limited and delayed host innate immune responses, facilitating bacterial survival and multiplication, but the resulting illness, tularemia, can progress to cause life-threatening sepsis [2]. At the cellular level, the bacterium is ingested by phagocytes but rapidly escapes the phagosome and replicates within the cytosol [3]. Among many characteristics of *F*. *tularensis* that may contribute to its ability to resist or delay triggering of innate host defenses are the synthesis and accumulation of free lipid A species that are very weak agonists of MD-2/TLR4 and expression of an O-antigen capsule [4, 5].

Early work on the virulence determinants of *Francisella tularensis* noted the presence of a thick electron-transparent outer structure that was believed to represent a putative capsule [6]. Subsequent studies noted that *Francisella tularensis* variants with a rough colony appearance, as opposed to the typical smooth one, possessed impaired virulence or were sensitive to serum [7]. Although these studies suggested the presence of a capsule, none had yet been isolated. More recently, Apicella *et al*. generated monoclonal antibodies from crude capsule extracts of *F*. *tularensis* subspecies *tularensis* and *holarctica* that recognized a high molecular weight (HMW) polysaccharide. One of these antibodies (clone 11B7) recognized a high molecular weight polysaccharide in immunoblots and localized to the surface of the bacterium as indicated by immunoelectron microscopy [4]. Using this antibody to guide further purification and structural characterization, they were able to determine that this HMW polysaccharide contained the identical four-sugar repeat found in the O-antigen of the organism’s lipopolysaccharide (LPS). The O-antigen capsule was not found in significant quantities in culture supernatants, suggesting that it was firmly attached to the bacterium [4]. Although free polysaccharide does not typically enter the gel during SDS-PAGE [8–10], the *Francisella* O-antigen capsule migrates slowly during SDS-PAGE, distinct from faster migrating, lower molecular weight LPS suggesting that the O-antigen capsular polymer contains a covalently attached lipid or protein. To address the possibility that the *F*. *tularensis* O-antigen capsule contains a covalently linked lipid moiety, we have in this study utilized metabolic radiolabeling of bacterial fatty acids synthesized during growth in broth culture to permit highly sensitive isolation and characterization of capsule-associated lipids [11].

We now report the presence of lipid covalently-linked to the O-antigen capsular polymers. The fatty acyl constituents recovered have the structural properties characteristic of *F*. *tularensis* lipid A, but with a composition distinct from the free lipid A that accumulates to high levels in Francisellae. These findings suggest that the O-antigen capsule is a specialized LPS molecule whose high-molecular weight sugar comprises an important surface feature of the bacterium. Further, the observation of distinct lipid A-like structures in the capsule suggests that the *Francisella* LPS biosynthetic pathway is somehow selective in determining which lipid A molecules are destined for decoration with capsule.

## Materials and Methods

### Materials and media

[1,2-^14^C] Acetic acid sodium salt (112 mCi/ mmol) was purchased from Moravek Biochemicals Inc. (Brea, CA, USA). Monoclonal Ab to *F*. *tularensis* LPS (clone FB11, catalog number 18742) was purchased from QED Bioscience, Ltd (San Diego, CA, USA). Sheep blood was purchased from Colorado Serum Co. (Denver, CO, USA). Bacto brain–heart infusion broth (BHI) was from Becton Dickinson (Sparks, MD, USA). Micrococcal DNAse was from New England BioLabs, Inc. (Ipswich, MA, USA). One-hundred percent ethanol (EtOH) was purchased from Deon Labs, Inc. (King of Prussia, PA, USA). PBS was from Mediatech, Inc. (Herndon, VA, USA). EconoSafe scintillation fluid was purchased from Research Products International (Mount Prospect, IL, USA). Sodium deoxycholate was from Sigma-Aldrich (St. Louis, MO). HPLC-grade grade chloroform (CHCl_3_) and methanol (MeOH) were obtained from Fisher Scientific (Pittsburgh, PA). HPLC-grade H_2_O was obtained from Sigma-Aldrich. Pyridine was obtained from Fisher Scientific.

*Francisella tularensis* subsp. *holarctica* strain LVS (ATCC strain 29684) was obtained from Dr. Michael Apicella. Stocks were frozen at -80°C and grown overnight (18 h) on cysteine heart agar with sheep blood at 37°C in 5% CO2. We supplemented BHI with [1,2-^14^C] sodium acetate, to a final concentration of 15 μCi/ml. Bacteria were suspended to an OD600 of 0.025–0.050 and incubated at 37°C with shaking at 200 rpm until the OD600 reached ~0.9 (approximately 8 h).

### Isolation of ethanol precipitate, capsule, and lipid A

Because ~60% of *Francisella* LPS is present as free lipid A, typical hot phenol and water isolation of LPS results in considerable losses of lipid A in the phenol phase [11]. Thus, a modified protocol was used to generate an ethanol precipitate (EtOHp) that contains the entirety of *Francisella* LPS and free lipid A (with some phospholipid contamination). Washed bacterial pellets were re-suspended to their original volume with 2% sodium dodecyl sulfate (SDS), 10 mM EDTA, 60 mM Tris base (pH 6.8) and incubated at 95°C for 5 min. The lysate was brought to 37°C and incubated overnight with proteinase K (Sigma-Aldrich), at a final concentration of 50 mg/ml. The treated lysate was diluted with three volumes of 100% EtOH and 1/10 volume of 3 M sodium acetate (pH 5.4) followed by incubation at -20°C overnight. LPS and nucleic acids were pelleted at 12,000 *g* at 4°C and washed twice in -20°C EtOH/sodium acetate as above. Recovered pellets were re-suspended in 50 mM Tris with 5 mM CaCl_2_ (pH 7.9) containing micrococcal nuclease (2000 Kunitz units/ml) and incubated overnight at 37°C.

### Isolation of high-molecular weight capsule polysaccharide

*Francisella* capsule was isolated by subjecting EtOHp to a modified hot phenol/water extraction followed by water/Triton X-114 partitioning and SDS gel filtration as described previously [4] (S1A Fig). An alternate protocol to isolate capsular polysaccharides from the nuclease-treated EtOHp (S1B Fig) was developed using deoxycholate (DOC)-based gel sieving [12]. EtOHp samples were dissolved in 2% DOC in 0.2 M NaCl, 50 mM Tris, 5 mM EDTA and sonicated for 30 min in a bath sonicator. After centrifugation to remove any insoluble debris, the sample was applied to a 16 mm x 30 cm Sephacryl S-200 column on an Akta Purifier FPLC system (GE Healthcare) at a flow rate of 0.5 ml/min. In order to remove any residual DOC that might interfere with subsequent chromatographic analysis, fractions underwent three rounds of ethanol precipitation as described above after addition of purified DNA (150 μg/ml UltraPure™ Salmon Sperm DNA Solution, Invitrogen) to enhance recovery. To control for any effects of the DOC or ethanol washing on subsequent chromatographic analysis, whole EtOHp was also treated in the same way in parallel with isolated capsule or low molecular weight material. Washed samples were raised in water and stored at 4°C until further analysis.

### Weak acid treatment

Release of lipid A from lipo-glycopolymers by selective cleavage of lipid A-3-deoxy-D-manno-oct-2-ulosonic acid (KDO) linkage was achieved via mild acid hydrolysis in 12.5 mM sodium acetate and 1% SDS (pH = 4.5) at 100°C for 30 min [13]. Extraction of released lipid A was accomplished by converting the solution to a two-phase Bligh-Dyer mixture by adding enough CHCl_3_ and MeOH to bring the hydrolysate to 2:2:1.8 (CHCl_3_:MeOH:water; v/v/v). After vortexing and a 1 h incubation at room temperature, the CHCl_3_ phase containing the released lipid A was removed. An equal volume of pre-equilibrated organic phase (from 2:2:1.8, CHCl_3_:MeOH:H_2_O) was then added to the remaining aqueous phase. After another 1 h incubation, this CHCl_3_ phase was combined with the first and then washed by adding pre-equilibrated aqueous phase. After vortexing and incubating for 1 h, the organic phase was removed. Organic phase samples were stored at -20°C until analysis.

### Sodium dodecyl sulfate-polyacrylamide gel electrophoresis

Samples were dissolved in lithium dodecyl sulfate sample buffer (Invitrogen, Carlsbad, CA, USA) and applied to a 4–12% Bis-Tris gradient gel in 2-(N-morpholino)ethanesulfonic acid buffer (Invitrogen), after which samples were transferred to polyvinylidene difluoride (PVDF) membranes (Millipore Immobilon® FL PVDF Membrane, LiCor Biotechnology, Lincoln, NE). For immunoblotting, the membrane was blocked in 5% dried-milk in Tris-buffered saline with 0.1% Tween-20 (pH 7.5) prior to incubation with mouse anti-*F*. *tularensis* LPS mAb (clone FB11) or mouse anti-*F*. *tularensis* capsule (clone 11B7 [4]). Primary Ab was detected using donkey anti-mouse fluorescent antibody (Li-Cor Biotechnology) and imaged using an Odyssey FC Imager (Li-Cor). For imaging by autoradiography, the membrane was dried and subjected to image analysis (PhosphorImager; Molecular Dynamics, Sunnyvale, CA, USA) using a tritium screen (GE Healthcare Life Sciences, Pittsburgh, PA) that permitted quantitation of as little as ~100 cpm/band of ^14^C-labeled material after 24 h of exposure.

### Thin-layer chromatography (TLC)

Fatty acids of intact bacteria and of various derived fractions were prepared for analysis by chemical hydrolysis using sequential treatment with 4 N HCl and 4 N NaOH at 90°C to release ester- and amide-linked fatty acids from the parent lipids, followed by neutralization and Bligh–Dyer lipid extraction [14]. The released free fatty acids were recovered in the lower (organic) phase. Normal-phase TLC was performed on 0.25-mm HPTLC silica gel 60 (EMD Millipore, Billerica, MA, USA) using hexane/ ethyl acetate/glacial acetic acid (50:50:1 v/v/v) as the solvent system [15]. Reverse-phase TLC used 0.2 mm high-performance-TLC (HPTLC Silica Gel 60, RP-18; EMD Millipore) with acetonitrile/acetic acid (1:1 v/v) as the solvent system [15]. Isolated lipid A species were resolved using normal-phase HPTLC (Silica gel 60, RP-18, Millipore, Billerica, MA) in a solvent system optimized to resolve *Francisella* lipid A species: CHCl_3_:Pyridine:88% Formic Acid:MeOH:H_2_O (54:46:16:0:5 v/v). Image analysis was performed using a tritium screen as described above.

### Reverse-phase high-performance liquid chromatography (HPLC)

Hydrolyzed samples containing free fatty acids were dried under nitrogen and dissolved in 2:1 CHCl_3_:MeOH. Samples were applied to a 5μ Grace Prevail organic acid 10 mm x 250 mm column at a flow rate of 1 ml/min on a Waters Alliance e2695 separations module (Waters, Inc. Milford, MA) and collected in 0.25 ml fractions. Samples were eluted in 85% mobile-phase A and 15% mobile phase B for 30 min, where A consisted of MeOH with 0.5% acetic acid and B of water with 0.5% acetic acid. The mobile phase was then changed to 100% A for 20 min. Total cpm in aliquots of various fractions was measured in a Beckman LS 5000TD liquid scintillation counter (Beckman Instruments, Inc., Fullerton, CA, USA). Recoveries typically exceeded 75%.

### Data presentation and statistics

Statistical tests were performed using Prism 6 (GraphPad Software, San Diego, CA, USA). Graphs were created with DataGraph 3 (Visual Data Tools, Inc., Chapel Hill, NC, USA), and compiled and annotated with OmniGraffle Pro 6 (Omni Group, Seattle, WA, USA).

## Results

### Identification of lipid A-like lipid associated with HMW O-Ag capsule of *Francisella* LVS

**Fig 1:**
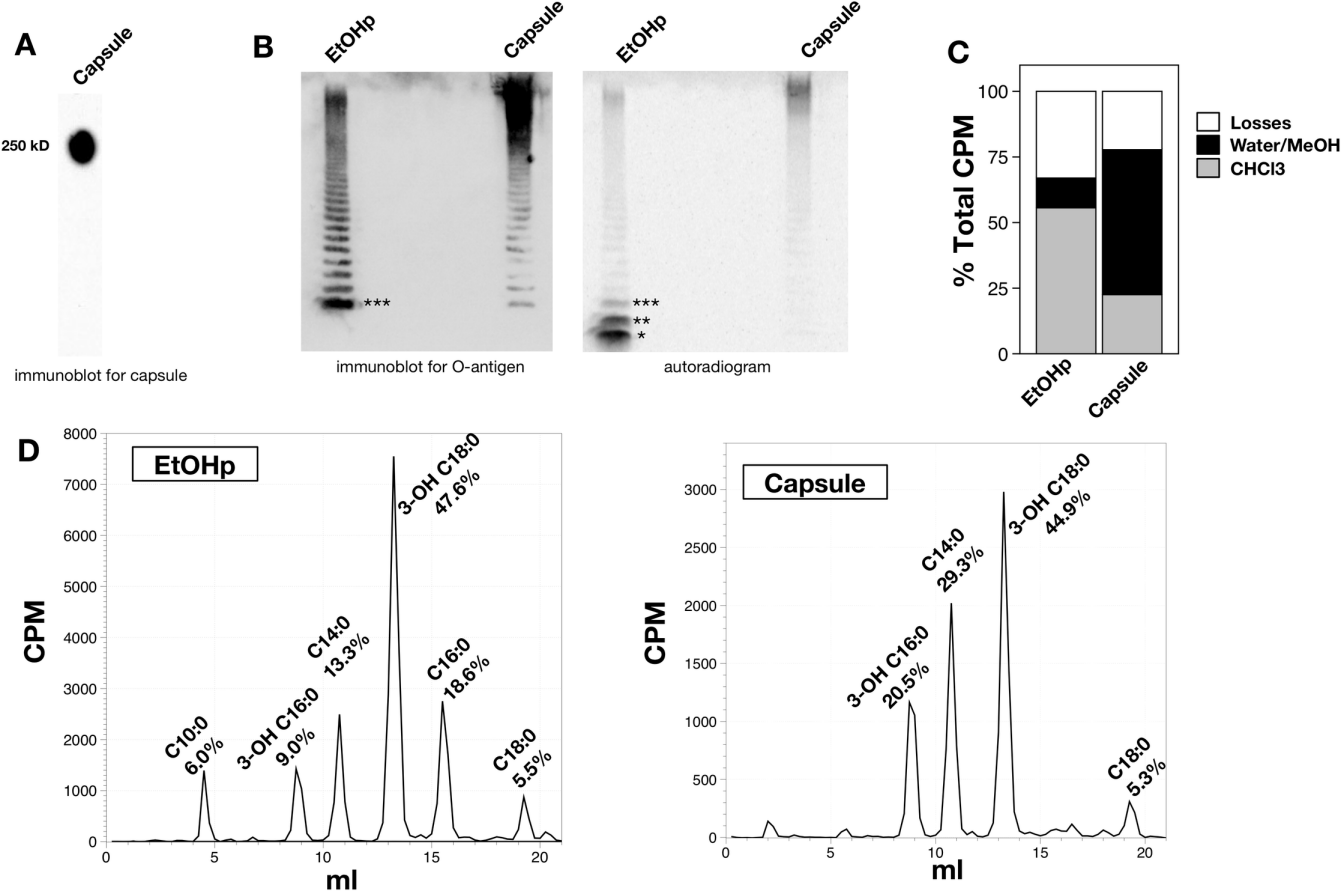
Analysis of O-antigen capsule prepared according to Scheme A, (S1A Fig) SDS-PAGE/immunoblotting for O-Ag capsule (antibody 11B7). The position of a 250 kD molecular weight standard is indicated. B) SDS-PAGE of metabolically labeled EtOHp and capsule, followed by immunoblot for *Francisella* O-antigen (clone FB11, left) or autoradiographic imaging (right). Denoted by asterisks are free lipid A (*), lipid A-core polysaccharide (no O-Ag) (**), and lipid A-core polysaccharide-single O-Ag unit (***) [16]. C) Distribution of cpm after chemical hydrolysis of EtOHp and capsule to release fatty acids. D) HPLC analysis of released [^14^C]-fatty acids from EtOHp and capsule extracted and recovered in the CHCl_3_ phase after chemical hydrolysis. Recovery of each individual fatty acid species is expressed as % of total recovery of [^14^C]lipids. Individual fatty acids were identified by comparison to commercial standards (C14:0, C16:0) or LC-MS analysis, as described previously [11].

To facilitate detection and characterization of lipid constituents of *Francisella* O-antigen capsule, we metabolically labeled bacteria during growth by supplementing BHI broth with 15 μCi/ml [^14^C]sodium acetate [11]. Initially, capsule was isolated from radiolabeled bacteria (28 million cpm from approximately 7 ml of broth culture) as described previously, by proteinase K digestion, extraction with hot phenol/water, and further enrichment of the capsule recovered in the aqueous phase by water/Triton X-114 partitioning and gel filtration (S1A Fig) [4]. SDS-PAGE and immunoblot revealed the presence of the high molecular-weight O-antigen capsule (Fig 1A, antibody 11B7). For comparison with the isolated capsule, we also analyzed the ethanol precipitate recovered after proteinase K digestion, which contains the entirety of *Francisella* LPS molecules (including free lipid A, which comprises ~60% of the total lipid A accumulated by Francisellae) (S1B Fig) [11]. Immunoblot of capsule for *Francisella* LPS O-antigen (O-Ag; FB11 antibody) confirmed the enrichment of higher molecular weight (HMW) polymers containing O-Ag and markedly diminished amounts of lower molecular weight LPS species containing O-Ag vs. EtOHp (Fig 1B, left panel). Examination of these same fractions by autoradiography demonstrated metabolic labeling of the HMW O-Ag capsular polymers and enrichment for higher molecular weight LPS and depletion of free lipid A (Fig 1B, right panel).

Since the O-Ag of *F*. *tularensis* contains acetylated sugars, further analysis was needed to definitively identify metabolically labeled lipids (e.g., fatty acids) within the HMW O-Ag polymers. This was accomplished by sequential 4N HCl/4N NaOH treatments at 90°C to release ester- and amide-linked fatty acids, if present, from the O-Ag polymers followed by Bligh/Dyer extraction to separate radiolabeled sugars from fatty acids in the water/MeOH vs. CHCl_3_ phases, respectively. Comparison of the recovery of radiolabeled material in the water/MeOH vs CHCl_3_ phases from capsule vs. EtOHp showed a much higher fraction of the recovered radioactive material from capsule (vs. EtOHp) in the H_2_O/MeOH phase, consistent with the high degree of enrichment of HMW O-Ag polymers in the capsule fractions (Fig 1C). Further characterization of the [^14^C]-labeled material recovered in the CHCl_3_ phase by HPLC (Fig 1D) revealed [^14^C] fatty acids that are grossly similar to those present in *F*. *tularensis* lipid A, which contains three 3-OH fatty acids and one nonhydroxylated fatty acid [5]. Fatty acids recovered from the EtOHp, which contains free lipid A in addition to lipid A-core and lipid A-core-O-Ag [11], were comprised primarily of the expected 3-OH-fatty acids, 3-OH-18:0 and 3-OH-16:0 (Fig 1D, left). The ratio of cpm in 3-OH C18:0 to that in 3-OH C16:0 in the EtOHp was 5.3, corresponding to a molar ratio of 4.7, consistent with the ratio previously reported for *Francisella* lipid A of 5–7:1 [11, 17, 18]. However, both the ratio of cpm in 3-OH-18:0/3-OH-16:0 (2.2) and the greater prominence of C14:0 and near absence of C16:0 distinguish the fatty acids linked to HMW O-Ag capsule (Fig 1D, right) vs. those present in the EtOHp. Taken together, these findings strongly suggest that the HMW O-Ag polymers that comprise the *F*. *tularensis* capsule contain lipid that closely resembles but is compositionally distinct from the more abundant free lipid A accumulated by these bacteria.

### Purification of HMW O-Ag capsule purified from EtOHp by deoxycholate-based gel sieving

**Fig 2:**
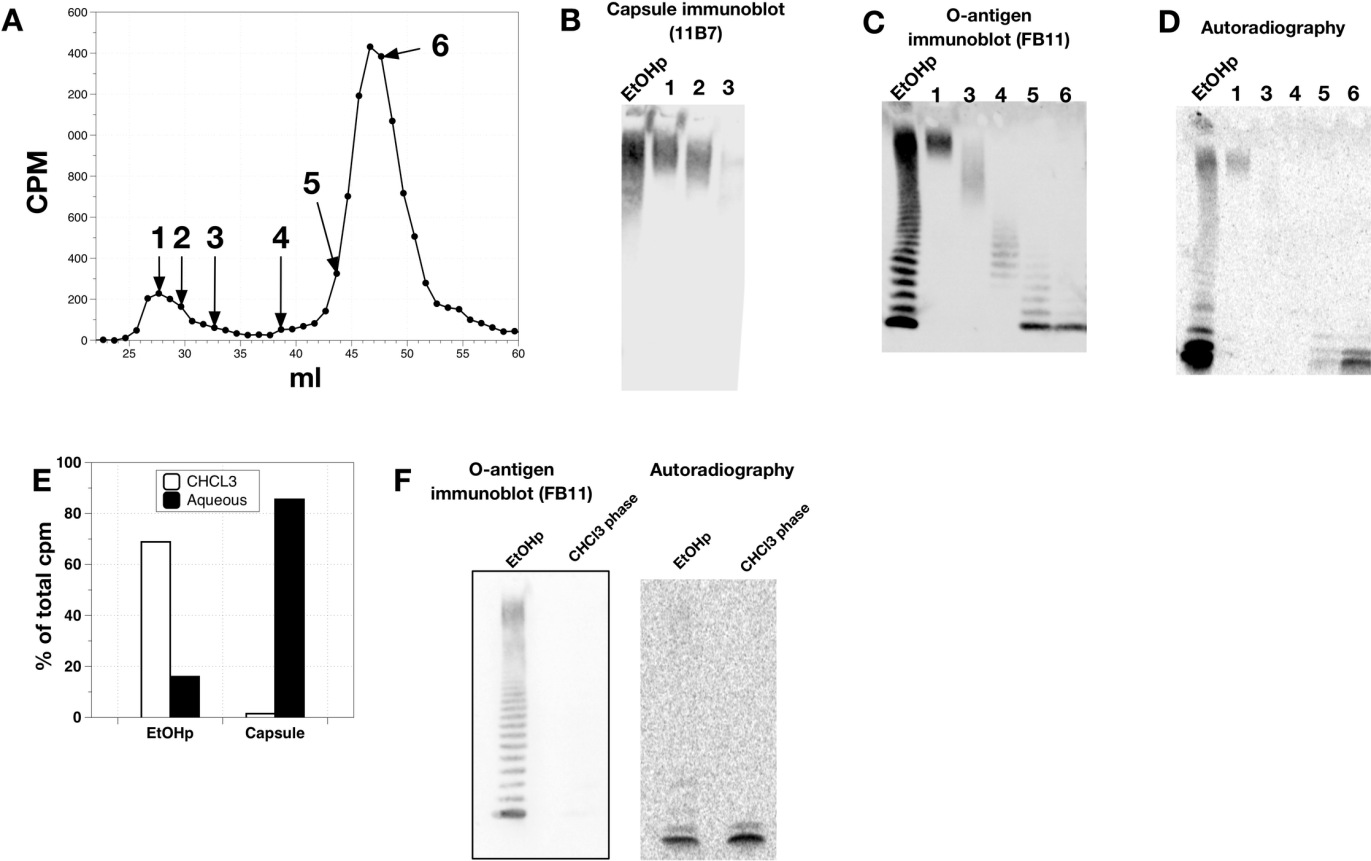
Deoxycholate/Sephacryl S-200-based gel sieving of EtOHp to isolate capsule. A) Results of gel sieving of the EtOHp. Recoveries of loaded cpm were typically ~100%. Fractions used for subsequent SDS-PAGE analysis are numbered. B) Capsule (11B7) immunoblot, C) O-antigen (FB11) immunoblot, and (D) autoradiogram, respectively of the indicated fractions. Data shown are representative of at least 4 independent experiments. E) EtOHp and capsule were subjected to Bligh-Dyer extraction and the partitioning of cpm is indicated. Numbers do not add up to 100% due to losses. F) O-antigen immunoblot and autoradiogram of EtOHp and of the extracted lipid fraction following Bligh-Dyer extraction of EtOHp.

To strengthen this conclusion, we utilized a second independent method to resolve and isolate HMW O-Ag capsule from lower molecular weight LPS. For this purpose, we adapted a deoxycholate (DOC)-based gel sieving system [12] to resolve metabolically labeled species in the EtOHp, obviating the need for hot phenol/water extraction, Triton X-114 partitioning, and SDS-based gel sieving. Radiolabeled EtOHp was sonicated in sample buffer (2% DOC in 0.2 M NaCl, 50 mM Tris, 5 mM EDTA) to maximize dispersion of sample in detergent and then applied to a Sephacryl S-200 column (16 mm x 30 cm) as described in the Materials and Methods. Quantitation of the cpm in individual fractions revealed a relatively small proportion of cpm in the highest molecular weight species (~9% eluting prior to 31 ml) and a large proportion of cpm that eluted as smaller molecules, consistent with the predominance of free lipid A and lipid A-core ± LMW O-Ag in *Francisella* (Fig 2A). SDS-PAGE and immunoblotting of the HMW species revealed expected isolation of HMW material reactive with the 11B7 capsule antibody (Fig 2B) and FB11 O-Ag antibody (Fig 2C), with no detectable radiolabeled lower molecular weight LPS species or free lipid A (Fig 2C and 2D). Conversely, the later eluting peak (i.e., fractions 44–53) was devoid of the HMW O-Ag capsule polymers and instead contained abundant free lipid A and lesser amounts of lipid A-core and LPS species containing relatively short polymers of O-Ag. Thus, DOC-based gel sieving of the EtOHp provided an even better isolation of the HMW O-antigen capsule while obviating the need for multiple 2-phase extractions.

### Lipid associated with HMW O-Ag capsule is covalently linked

In order to confirm that isolated HMW O-Ag rich fractions were not contaminated with the free lipid A that is so plentiful in LVS, we subjected HMW capsule to Bligh-Dyer lipid extraction. As expected, a large majority of cpm of the EtOHp partitioned to the organic phase (Fig 2E), which when analyzed by SDS-PAGE and immunoblot for FB11 and autoradiography was greatly enriched for the two fastest-migrating bands corresponding to free lipid A and lipid A + core, with virtually no contaminating O-antigen (Fig 2F). In contrast to the EtOHp, virtually none of the capsule cpm (1.5%) were found in the CHCl_3_ phase (Fig 2E). These results confirm that the capsule fractions resolved by DOC-based gel sieving are free of significant quantities of free lipid, and thus, the lipid A-like material recovered is covalently linked to the HMW O-Ag capsule.

### Fatty acid composition of lipid linked to HMW O-Ag capsule differs from that of the overall EtOHp

**Fig 3:**
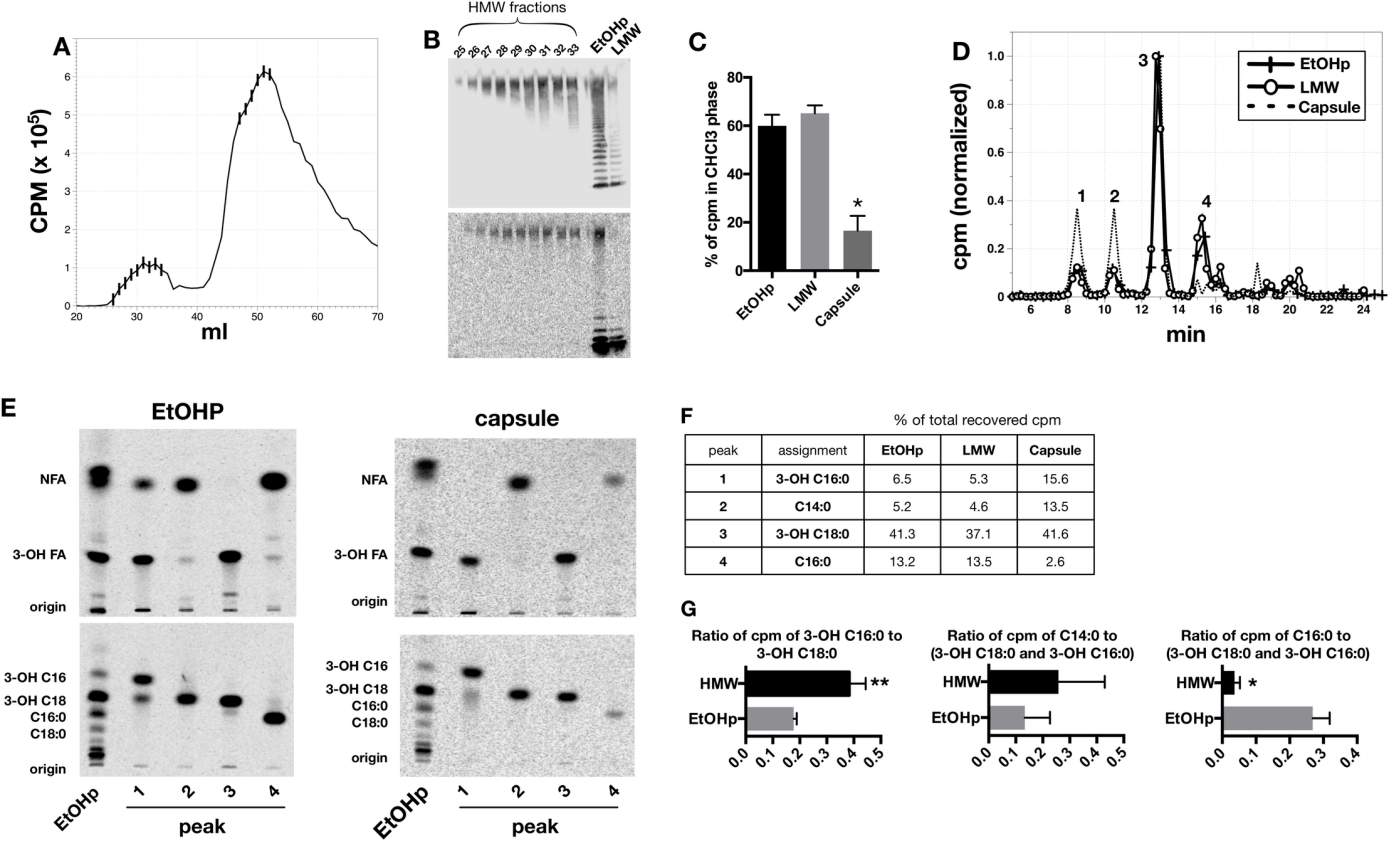
Fatty acid analysis of HMW O-Ag capsule. A) Elution of [^14^C] labeled species during deoxycholate/Sephacryl S-200 chromatography of radiolabeled EtOHp (larger sample input). B) SDS-PAGE followed by immunoblot (O-Ag; FB11, top pane) and autoradiogram (bottom pane) representing capsule- and LMW-rich species (fractions 25–33 and 46–51, respectively). C) Percent of cpm of the indicated samples recovered in the CHCl_3_ phase after chemical hydrolysis and Bligh-Dyer extraction are indicated. * denotes that recovery from capsule is significantly lower than that from both EtOHp and LMW samples, per Tukey’s test for multiple comparisons (*P* < 0.001). D) HPLC profile of recovered [^14^C] species in CHCl_3_ phase from EtOHp, nearly O-Ag-free LMW species, and HMW O-Ag capsule. Each curve was normalized so that the peak of the most abundant species (peak 3) was set at 1.0. E) Normal-phase TLC (top panels) and reverse-phase TLC (bottom panels) of the 4 major peaks derived from EtOHp (left) and capsule (right). *Francisella-*derived and purified commercial FFA were used to identify individual fatty acids, as indicated. NFA, non-hydroxylated fatty acids. 3-OH FA, 3-OH fatty acids. Note that C14:0 and 3-0H-18:0 (peaks 2 and 3) closely migrate on reverse-phase TLC but are readily distinguished by normal phase TLC. F) Relative content of the 4 major FA substituents of the EtOHp, LMW, and capsule samples, expressed as percent of total cpm recovered. These results represent analyses of fractions derived from the same population of metabolically labeled bacteria. G) Similar analyses were performed on three separate EtOHp and capsule preparations from independent batches of labeled bacteria to demonstrate that FA compositional differences between the EtOHp (in which free lipid A is most abundant) and HMW O-Ag capsule are reproducible and, where indicated (*, *P* < 0.05 and ** *P* < 0.001 for paired T tests) significant. *Left* panel: ratio of [^14^C] 3-OH-16:0 to [^14^C] 3-OH-18:0; *center* panel: ratio of [^14^C] C14:0 to combined [^14^C] 3-OH-16:0 + 3-OH-18:0; *right* panel ratio of ^14^[C] C16:0 to combined [^14^C] 3-OH-16:0 + 3-OH-18:0.

DOC-based gel sieving was scaled-up nearly ten-fold to provide sufficient material for further analysis of capsule-associated lipids. EtOHp was isolated from 56 ml of metabolically radiolabeled late-log LVS cultures and subjected to gel sieving (Fig 3A). Fractions eluting near the void volume were subjected to SDS-PAGE followed by immunoblotting for O-antigen (Fig 3B, top) and autoradiography (Fig 3B, bottom) to confirm enrichment for HMW O-Ag polymers. These fractions were combined as were later-eluting fractions, which were enriched in free lipid A and lipid A-core polysaccharide ± short polymers of linked O-Ag (pooled fractions, 46–51, "LMW"; Fig 3B). The unfractionated EtOHp was analyzed in parallel after dispersal in DOC sample buffer to control for any effects of residual detergent on the subsequent analysis. The two pooled samples (i.e., HMW capsule and LMW) and the EtOHp were subjected to sequential 4N HCl and 4N NaOH treatments at 90°C followed by Bligh/Dyer extraction to recover released lipids in the CHCl_3_ phase. A significantly smaller fraction of the total cpm of the treated capsule (vs. EtOHp and LMW) was recovered in the CHCl_3_ phase (Fig 3C), consistent with the increased abundance of metabolically radiolabeled acetyl sugars of the polar polysaccharide chains of the HMW O-Ag capsule polymers [4, 19].

HPLC of the recovered radiolabeled compounds from the respective CHCl_3_ phases yielded from each sample several peaks (major peaks 1–4, Fig 3D) which were further analyzed by both normal- and reverse-phase TLC to confirm the identity of the fatty acids present in each peak (Fig 3E). These analyses confirmed that the major 3-OH-fatty acid constituents of free and LPS-linked lipid A (3-OH C18:0 and 3-OH C16:0) were also the most prominent components of capsule-associate lipids. However, as originally observed in HMW O-Ag rich preparations obtained by Triton X-114 partitioning and SDS-based gel sieving (Fig 1), the molar ratio of 3-OH C18:0 to 3-OH C16:0 was much less in capsule than either the starting material (EtOHp) or the pooled LMW fractions (2.7 for capsule vs. 6.4 for EtOHp vs. 7.1 for LMW). In addition, the most abundant non-hydroxylated FA constituent differed significantly between HMW O-Ag capsule (mainly C14:0) and pooled fractions comprising free and LPS-linked lipid A (mainly C16:0, Fig 3D and 3F). When results from three capsule isolations were analyzed and compared to that of the three EtOHp preparations from which the purified HMW O-Ag capsule was derived, the compositional FA differences were again observed, including: increased presence in capsule of 3-OH-16:0 (Fig 3G, left; p<0.05) and C14:0 (Fig 3G, center, did not reach statistical significance) and decreased presence of C16:0 (Fig 3G, right; p<0.001).

### Release of lipid-linked to HMW O-Ag capsule by mild acid treatment

**Fig 4:**
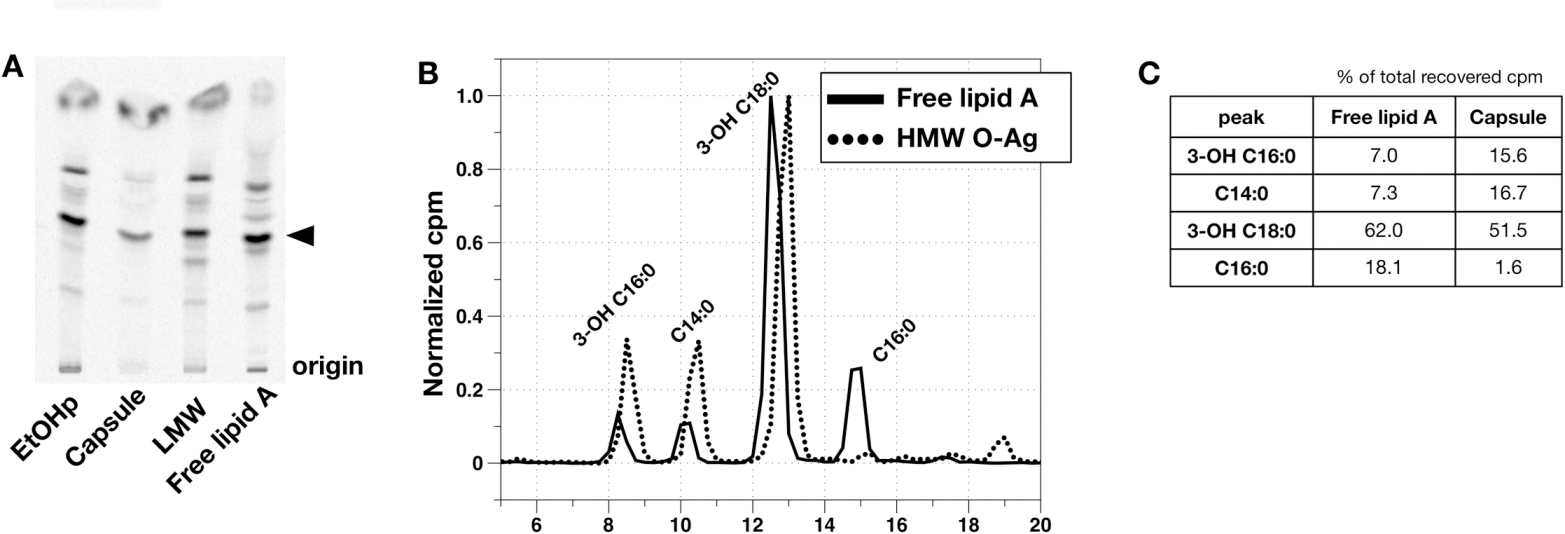
Fatty acid analysis of lipid released from HMW O-Ag capsule by mild acid treatment. A) HPTLC of lipids recovered after mild acid hydrolysis of the indicated preparations. The solvent system used was CHCl_3_:Pyridine:88% Formic Acid:MeOH:H_2_O (54:46:16:0:5 v/v). The arrowhead indicates the dominant species in free lipid A. Highly enriched free lipid A was derived via Bligh-Dyer extraction of the EtOHp (see Fig 2F). B) HPLC analysis of [^14^C] fatty acids derived from free lipid A and lipid released by mild acid treatment of HMW O-Ag. C) The four major peaks in B were quantified and expressed as the % of total recovered cpm.

Lipid A is bound to core ± O-Ag polysaccharide via a relatively labile ketoside bond with 3-deoxy-D-*manno*-oct-2-ulosonic acid (KDO) and can be released after mild acid hydrolysis (in 1% SDS at a pH of 4.5 [13]). To test if the lipid linked to the HMW O-Ag capsule has similar properties, the metabolically radiolabeled HMW O-Ag capsule was treated in the same way. After subjecting capsule to this treatment, approximately 14% of total capsule cpm partitioned to the CHCl_3_ phase, with the majority remaining in the aqueous phase. The radiolabeled lipid liberated by mild acid treatment was then analyzed using an HPTLC system optimized for *Francisella* lipid A [11]. The major lipid released from the HMW capsule migrated like the major species present in free lipid A and in LMW and EtOHp samples that had been subjected to mild acid treatment (Fig 4A). Fatty acid analysis of the lipid liberated from HMW O-Ag capsule by mild acid treatment (Fig 4B and 4C) revealed a composition similar to that of the untreated HMW O-Ag capsule (Fig 3F), indicating that the lipid released by mild acid treatment was representative of the major lipid associated with the HMW O-Ag capsule.

## Discussion

*Francisella tularensis* produces a HMW O-antigen capsule that is stably associated with the cell surface and not shed in significant quantities into broth culture supernatants ([4] and data not shown). Our results indicate that HMW O-Ag polymers isolated from *Francisella* LVS contain a covalently linked lipid A-like molecule. Evidence in support of this conclusion includes the prominence in highly purified HMW O-Ag polymers of 3-OH-fatty acids, unique constituents of lipid A in bacteria [20], and, more specifically, 3-OH-18:0 and 3-OH-16:0 that are the signature fatty acids of *Francisella* lipid A and LPS (Figs 3D & 3E; 4B & 4C; and [5, 21, 22]). The presence of these fatty acids in the purified HMW O-Ag polymers even after various treatments (e.g., hot phenol/water or Bligh-Dyer extraction; Triton X-114 partitioning and/or SDS- or DOC-treatment) known to dissociate free lipid A from complex samples [4, 13, 16] strongly suggests a covalent linkage. Further evidence that the covalently-bound lipid is lipid A is provided by the recovery from HMW O-Ag polymers of a majority of these lipid(s) by mild acid-SDS treatment designed to liberate lipid A that is linked to a (poly)saccharide via a relatively labile ketoside bond with KDO. Finally, the similar migration during TLC of this released lipid in comparison to the major lipid A species of free lipid A and LPS-linked lipid A (Fig 4A) is consistent with its identity as a lipid A-like molecule.

Remarkably, despite the close resemblance of the lipid(s) linked to the HMW O-Ag polymers to either free or LPS-linked lipid A, our findings indicate that the HMW O-Ag polymer-linked lipid(s) are structurally distinct. The evidence supporting this conclusion is principally two-fold: i) the difference in the molar ratio of 3-OH-18:0/3-OH-16:0 (ca. >5 in free lipid A/LPS vs. <3 in the HMW O-Ag polymers (capsule); Figs 3G and 4C); and ii) the marked difference in the most prominent non-hydroxylated fatty acid present (mainly C16:0 in free lipid A/LPS, C14:0 in HMW O-Ag capsule (Figs 3G and 4C). The molar ratio of 3-OH-18:0/3-OH-16:0 and prominence of C16:0 we measured in metabolically labeled total free lipid A + LPS correspond closely to published data on the fatty acyl composition of *Francisella* lipid A [5, 17, 18, 21, 22]. Hence, the unusual compositional features of the lipid A-like molecule(s) linked to HMW O-Ag polymers appear to be a unique structural characteristic of these polymers. The near absence of C16:0 in the HMW O-Ag polymers is particularly striking given the prominence of this non-hydroxylated fatty acid in all the mass spectroscopic analyses of *Francisella* lipid A reported to date. Taken together, these compositional analyses strongly suggest that the lipid A-like molecule linked to HMW O-Ag polymers is structurally distinct from the bulk lipid A in *Francisella* present as free lipid A. Other structural differences (e.g., involving charged and/or uncharged polar substituents of lipid A), as well as heterogeneity of capsule-associated lipid, are possible but will require purification of much greater amounts of the HMW O-Ag polymers and liberated lipid for more definitive structural analyses.

The Francisellae produce a remarkable variety of lipid A structures, including multiple isobaric species with varied fatty acid substitution patterns [21, 22]. Analyses of whole bacterial LPS demonstrate the presence of minor populations of lipid A containing both 3-OH and non-hydroxylated fatty acids of varying acyl chain length. Among these are minor species containing the shorter C14:0 non-hydroxylated fatty acid that we find enriched in the HMW O-Ag capsule [21, 22]. In part, this compositional diversity could reflect promiscuous fatty acid substrate properties of the various *Francisella* lipid A acyltransferases. In addition, as exemplified by the differential temperature-dependent activity and expression of two *lpxD* genes that possess distinct fatty acid selectivity, lipid A/LPS fatty acid composition can be modulated in response to changing environmental conditions [23].

**Fig 5:**
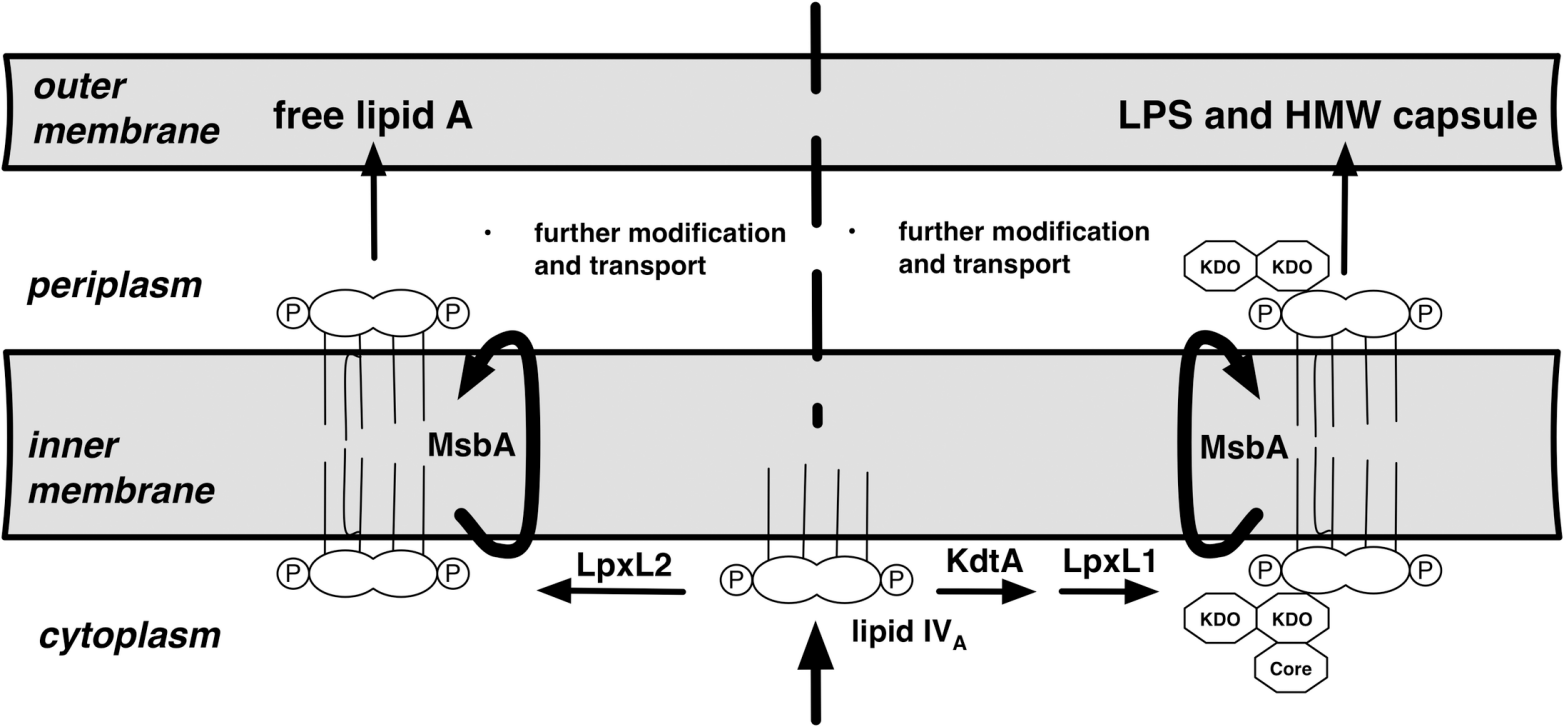
Hypothesis of possible mechanistic bases of distinct fatty acid composition of free lipid A vs. lipid A linked to the HMW O-Ag capsule in *F*. *tularensis*. Lipid IV_A_ contains 4 mol of 3-OH fatty acid per mol of lipid A (3-OH-18:0 >>3-OH-16:0). LpxL1/2 are homologous "late" acyltransferases that subsequently incorporate the single non-hydroxylated fatty acid of *F*. *tularensis* lipid A. Based in part on Raetz et al., we hypothesize that only one (arbitrarily, in this figure, LpxL1) is KDO-dependent and selective for C14:0 whereas LpxL2 is KDO-independent and selective for 16:0 [25]. The different 3-OH-FA composition of free lipid A and lipid linked to HMW O-Ag capsule could reflect the substrate specificity of KdtA that incorporates KDO into lipid IVA.

Our finding of distinct lipid A structures in the free lipid A pool of *Francisella* vs. the HMW O-antigen capsule suggests a possible relationship between the fine structure of lipid A and the synthesis and accumulation of HMW O-Ag capsule. In the synthesis of LPS, attachment of the 3-OH-fatty acids to the (di)glucosamine backbone of lipid A precedes attachment of KDO (e.g., lipid IV_A_ in Fig 5) whereas subsequent incorporation of non-hydroxylated fatty acid(s) by "late" acyltransferases, trans-bilayer migration across the inner membrane, and transport to the outer membrane typically follow KDO attachment [24]. The remarkable abundance of free lipid A in the Francisellae suggests an unusual capacity of this Gram-negative bacterium to carry out both late acylation and envelope transport in a KDO-independent fashion. Raetz et al. have proposed that one of the two late acyltransferase genes in *Francisellae* is KDO-dependent, which raises the possibility that only a subset of lipid A is subject to modification by this enzyme ([25]; arbitrarily labeled as LpxL1 in Fig 5). Fatty acid preference of an individual late acyltransferase in *Francisella* has been suggested by the effects of mutation of one of these late acyltransferases (FTT02323c). Comparison of the lipid A composition of the wild-type and mutant strains revealed a selective loss in the mutant of a minor lipid A subspecies that is strikingly similar in fatty acid composition to the lipid A we have observed linked to HMW O-Ag capsule (1 mol C14:0, 2 mol 3-OH-18:0 and 1 mol 3-OH-16:0) [26]. Apparently in its place, the mutant contained a novel triacyl species, identical to the missing species except for the absence of a myristate. We hypothesize that the selectivity of this late acyltransferase for KDO-containing lipid A could account at least in part for the predominance of C14:0 in the HMW O-Ag capsule (Fig 5). Additionally, the different 3-OH-FA composition of free lipid A vs. lipid A linked to HMW O-Ag capsule could reflect the substrate specificity of KdtA that incorporates KDO into lipid IVA. Whether the lipids associated with lower MW LPS resemble the HMW O-Ag capsule-associated lipid is unknown. Once better separation can be achieved of the abundant free lipid A from short and intermediate-length LPS, it should be possible to determine which structural features are associated with all KDO-containing LPS vs. only HMW O-Ag capsule.

Ligation of O-Ag to lipid A-core polysaccharide takes place on the outer leaflet of the inner membrane [24], before transport of LPS to the outer membrane. We speculate that synthesis and transport of the HMW O-Ag capsule to the surface of *Francisella* similarly requires ligation of the O-Ag to a lipid A-core polysaccharide acceptor on the outer leaflet of the inner membrane. In support of this speculation, the *Francisella* O-Ag capsule seems to most closely resemble the group 4 capsules originally characterized in *Escherichia coli* and classified by Whitfield [27]. Like group 4 capsules, the *Francisella* O-Ag capsule is composed of O-Ag repeats containing acetamido sugars and is dependent upon the Wzy system for polysaccharide polymerization [28, 29]. Mutations in *Francisella* core LPS synthetic enzymes and in the O-Ag ligase markedly impair O-Ag capsule production [28, 29], further suggesting that the LPS synthetic machinery is utilized for HMW O-Ag capsule synthesis. Export of LPS to the outer membrane is carried out by the LPS transport proteins LptABCDEFG [30] that include multiple proteins purported to bind specifically to the lipid A portion of LPS [31–33]. If either binding and/or transport efficiency is selective for lipid A of a particular fatty acid composition, the enrichment in HMW O-Ag polymers of lipid A species of shorter chain 3-OH and non-hydroxylated fatty acids could reflect those species that are transported less efficiently, hence allowing more time for synthesis and attachment of the longer O-Ag polymers characteristic of this capsule (Fig 5).

The FB11 mAb used to detect the O-Ag of *Francisella* LPS and HMW O-Ag capsule recognizes the terminal O-Ag unit of each polymer [34]. Thus, each species of differing O-Ag chain length is recognized with similar affinity on immunoblots, providing an estimate of the relative molar abundance of the various O-Ag polymers. Such analyses of the EtOHp fraction that contains nearly all the free and LPS-linked lipid A of *Francisella* as well as the HMW O-Ag polymers (Figs 1B and 2C and [11]) reveal a bimodal distribution, with the most abundant species being 1) LPS with relatively few O-Ag repeats, and 2) the HMW O-Ag capsular polymers that are recognized by their selective reactivity with the anti-capsular 11B7 mAb (Fig 2B). The selectivity of the 11B7 mAb for the HMW O-Ag polymers could reflect the presence of a unique conformational epitope within the HMW O-Ag polymers or the presence of other features independent of the O-Ag repeats. Alternatively, the selectivity could be the result of low-affinity interactions with an internal epitope of the O-Ag repeat that requires the abundant presence of this epitope in the longest O-Ag chains to produce a detectable reaction [35]. Whatever the basis of the selective reactivity of the anti-capsular 11B7 mAb, its combined use with the FB11 mAb was key in demonstrating the efficacy of DOC-gel sieving in purification of the HMW O-Ag capsular polymers.

Unfortunately, attempts thus far to scale-up the gel sieving protocol to provide sufficient material for more complete structural analysis of the HMW O-Ag-linked lipid A have been frustrated by the viscous properties of the capsule that reduce chromatographic resolution at high sample loads and, possibly, reduce the release of the linked lipid A under the mild hydrolysis conditions required to recover lipid A for mass spectrometry analysis. The combined use of metabolic labeling with the fractionation of the HMW O-Ag polymers under the conditions described has made it possible to estimate the relative molar abundance of the HMW O-Ag polymers (vs. free and LPS-linked lipid A) and the apparent average chain length of the O-Ag repeats. Purified HMW O-Ag polymers represent <10% of the total radiolabeled material recovered following DOC-gel sieving, <20% of which is radiolabel derived from the lipid linked to the capsular polymers. Provided the lipid we have characterized linked to HMW O-Ag capsule is indeed a lipid A, these findings indicate that the lipid A linked to HMW O-Ag capsular polymers represent <~2% of the total lipid A produced and accumulated by growing *Francisella* under the conditions tested, underscoring the sensitivity of the methods used to reveal and partially characterize the lipid linked to the O-Ag capsular polymers. Based on the derived fatty acyl composition of the capsule-linked lipid A-like molecule and the number of acetylated sugars present in the O-Ag repeats, the distribution of radioactivity after sequential 4N HCl/4N NaOH treatments at 90°C and Bligh-Dyer extraction (Fig 3C) suggests an average chain length of >40 O-Ag repeats for the HMW O-Ag capsular polymers. It should be possible in the future to apply the same combination of methods and reagents under other bacterial growth conditions, including within infected human phagocytic cells [11], to test for regulation of *Francisella* LPS and O-Ag capsular synthesis and accumulation.

We hypothesize that the association of the HMW O-Ag capsule with a particular subset of lipid A contributes to the means whereby the organism regulates the amount of O-Ag and capsule expressed. Studies are underway to isolate short and intermediate-length LPS to determine if the novel lipid is also associated with these species. If shorter LPS species contain distinct lipid A structure from that of the more abundant free lipid A, then it is possible that the bacterium uses distinct lipid A structures to regulate the overall balance between free lipid A and core ± O-Ag-containing LPS/ HMW capsule. Given that two late acyltransferases appear to differ in their dependence for KDO in the accepting lipid A (Fig 5), we are generating mutants that ablate the synthesis of these enzymes in strains that we can readily study outside of a BSL3 lab using metabolic labeling (e.g. LVS and *F*. *novicida*). If downstream events like flipping lipid A to the periplasm or ligation of O-Ag are selective for lipid A with a particular fatty acid composition, then these mutants may contain altered proportions of O-Ag capsule or LPS overall. In sum, these strains could provide unique experimental tools in studies of the biogenesis of free lipid A, short chain LPS, and HMW O-Ag capsule and evaluation of their possible roles in the various stages of interactions of Francisellae with the host.

The LVS strain that we have described was derived from a *F*. *tularensis* subspecies *holarctica* strain in the 1950s [1]. Direct comparisons of the LPS structures from various subspecies of *F*. *tularensis* have shown that the major compositional features of the lipids A are similar [36], but include variations in the degree of substitution of lipid A with phosphate or hexoses [11, 37]. Although the LPS and HMW capsule of LVS is serologically identical to that of the more virulent strains such as Schu S4 (a subspecies *tularensis* isolate), it remains to be determined if the variations in lipid A structure we have observed are unique to LVS, or to the *F*. *tularensis* subspecies *holarctica*.

In conclusion, our results identify a lipid that is covalently-associated with HMW *Francisella* LVS O-Ag capsule. The presence of 3-OH fatty acids, which are unique to lipid A among bacteria, and liberation of this lipid by gentle acid treatment are consistent with it being a lipid A-like molecule. That the fatty acids found in this lipid are distinct from those that are predominant in the organism overall suggests that *Francisella* regulates HMW O-Ag capsule expression in a novel way.

## Supporting Information

S1 Fig Schema of methods used. A) Schematic representation of the method to isolate O-Ag capsule as described by Apicella et al. [4] B) Schematic of the method used herein to isolate EtOHp.
(TIFF)
