# Supplementary figures and images for: Evidence Suggesting That Francisella tularensis O-Antigen Capsule Contains a Lipid A-Like Molecule That Is Structurally Distinct from the More Abundant Free Lipid A
Source: PLoS One. 2016 Jun 21;11(6):e0157842. doi: 10.1371/journal.pone.0157842 (PMC4915664; doi:10.1371/journal.pone.0157842)

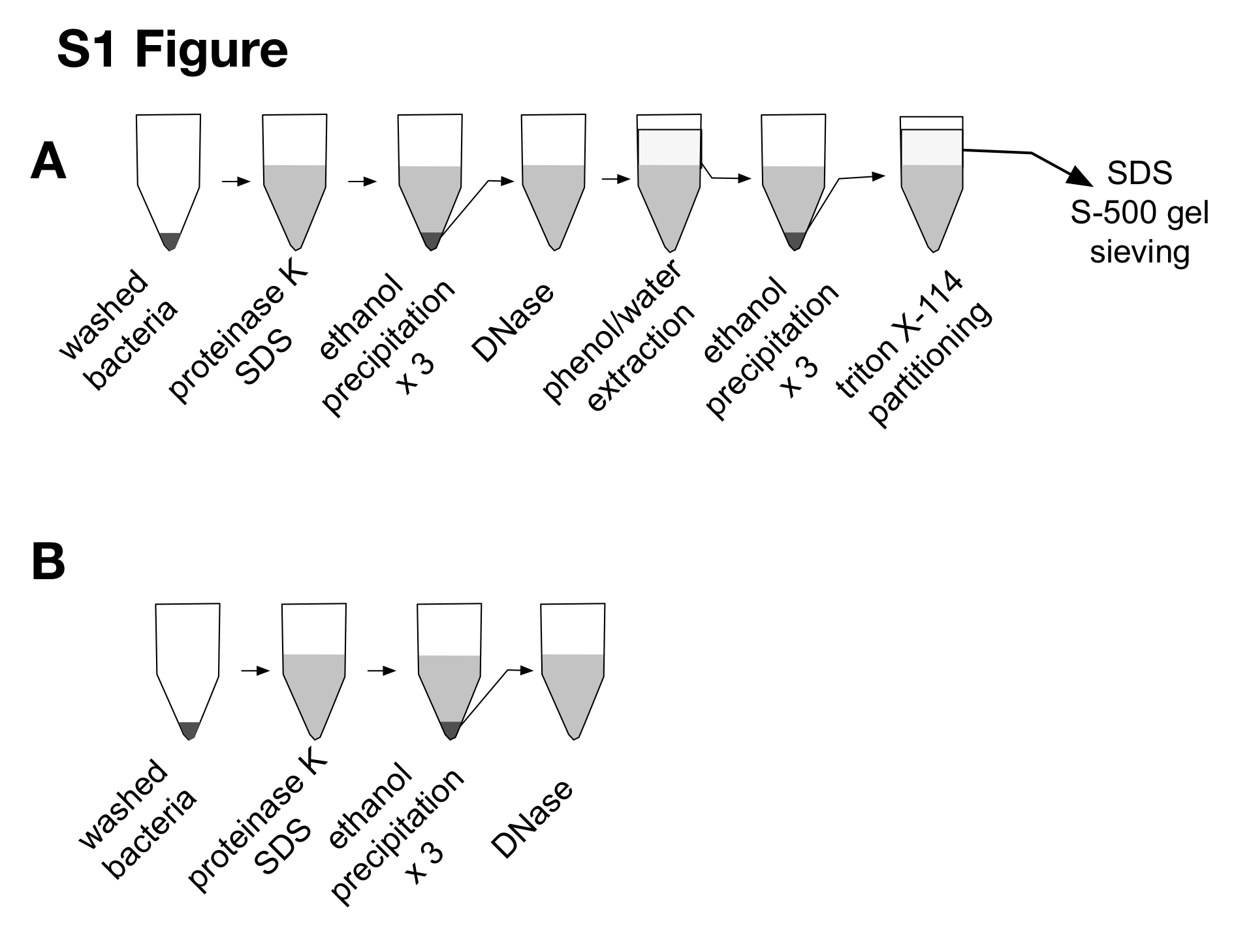

Supplement: S1 Fig — A) Schematic representation of the method to isolate O-Ag capsule as described by Apicella et al. [4] B) Schematic of the method used herein to isolate EtOHp. (TIFF) [file pone.0157842.s001.tiff]
